# Healthy Environments: Understanding Perceptions of Underrepresented Communities in the United Kingdom

**DOI:** 10.3390/ijerph19159643

**Published:** 2022-08-05

**Authors:** Lily F. Roberts, Olivia Lounsbury, Veronica Awuzudike, Neil Jennings, Emma L. Lawrance

**Affiliations:** 1 Institute of Global Health Innovation, Imperial College London, London SW7 2AZ, UK; 2 School of Public Health, Imperial College London, London SW7 2AZ, UK; 3 Grantham Institute, Imperial College London, London SW7 2AZ, UK; 4 Mental Health Innovations, London EC4Y 8JJ, UK

**Keywords:** healthy environment, public involvement, diversity and inclusion, mental health, mental wellbeing, nature, co-production

## Abstract

A healthy environment has been defined by global health organisations as one that is safe, supportive of healthy lifestyles, and free of hazards. Such definitions disregard the complexity of what it means for an environment to be perceived as ‘healthy’—such as the mental, not just physical, health effects on citizens. This study aimed to understand the attributes that underrepresented groups of the United Kingdom (UK) public assign to healthy environments—an important step for directing public policy and actions to create environments that are inclusive of all citizens. This co-created study involved 95 participants from underrepresented communities in 10 separate focus groups, each facilitated by a community member. Thematic analyses highlighted five key attributes of a healthy environment: sounds and sights, accessibility, safety, familiarity and mental health and wellbeing. This study draws a picture of key attributes underrepresented groups of the UK public assign to healthy environments that is richer than that drawn by existing definitions. These findings illustrate the importance of hearing diverse voices when directing research, policy, and actions that attempt to develop healthy environments for all.

## 1. Introduction

A healthy environment is a prerequisite to basic human rights, including the rights to life, health, food, water, and sanitation [1,2], and has been recognised as such by the United Nations as part of their 2030 Agenda for Sustainable Development [3]. A healthy environment, as defined by leading global and national health organisations—the World Health Organization, UNICEF, and the United States Center for Disease Control and Prevention—includes air, land and water that is safe for human health, supportive of healthy lifestyles, and free of hazards such as toxic chemicals which can reduce quality of life and create health problems [2,4,5]. This would suggest that, conversely, an unhealthy environment contains elements deemed unsafe for human health and quality of life, such as air or water pollutants or roads lacking appropriate pedestrian and cycling infrastructure to prevent injury when people are moving around. Such unhealthy environments exacerbate health inequalities, since exposures such as air pollution are more likely to affect more deprived communities and individuals within those communities with existing health problems [1]. These definitions are applicable to both natural and built environments, as both can promote or hinder good health [6]. As such, in its Healthy New Towns programme, the United Kingdom’s (UK) National Health Service (NHS) highlights the role of the built environment in wellbeing and health promotion [7]. Noticeably, these existing definitions concentrate on physical health impacts but fail to explicitly acknowledge associations between the environment and mental health and wellbeing—such as the effect of traffic noise on increasing depression risk [8] or the benefits of visiting urban green space and countryside to mental wellbeing [9]. Reducing the negative impacts and maximising the positive impacts of environments on mental wellbeing can increase community resilience and foster closer connections between people and place [10].

Recognising the essential role of healthy environments in healthy societies and optimising the environment to realise these benefits to human health and wellbeing should be top priorities for public policy. Characterising how different social groups understand the potential of the natural environment for human health and identifying the factors that may support or hinder realisation of these health benefits are research priorities for UK ministerial departments [11]. As Prüss-Ustün et al. advocate, “A change in perception to view the environment as an essential element of health protection, while adequately preserving it, would greatly benefit people’s health” [5]. However, despite its importance to the health and wellbeing of all citizens, the topic of a healthy environment remains somewhat inaccessible. The conceptualisation of healthy environments and ecosystems is seen as complex and abstract to the general public, and communication barriers exist between researchers, policy makers and the public [12]. These barriers can restrict meaningful discussion across these groups about the societal and systemic changes required to address the climate and ecological crises. It is well known that a rapid decarbonisation of our societal infrastructure is required to solve the climate crisis [13] and that such change will require rapid individual, community- and system-level action. Such climate action also needs to take place in a manner that does not adversely affect the ecological crisis. To achieve a Just Transition to a lower carbon future [14,15], it is essential that decision-makers direct policies and allocate resources in a manner that reflects public priorities—and that the priorities of all groups within society are carefully considered. Open dialogue about these issues can be stymied by lack of appreciation and awareness of the perceptions of different groups to what they believe society should be aiming for; and what constitutes a healthy environment for people and planet. Accommodating the perspectives of seldom heard community groups can facilitate climate narratives and policies that are more inclusive and appreciative of the range of understandings of what constitutes a healthy environment.

Social and environmental factors are influential for health and wellbeing, but will “vary in importance between communities, because of differences in the current services, facilities, priorities and needs of the communities, and because communities change over time” [16]. The successful design and implementation of environmental features that support climate action—such as cycle lanes and urban green spaces—depends on dialogue between policy makers, planners and community members to ensure these features align with communities’ needs and waste is minimised [6]. Yet such collaborations are often hampered by implicit bias and poor accessibility of environments such as green spaces to certain groups of society [17,18]. As such, certain communities have been found to spend less time outdoors than others, including Black, Asian and minority ethnic groups (BAME), urban-dwellers, adolescents, people with disabilities, and people living in deprived areas [19,20,21]. It is imperative to have an informed understanding of how a range of community groups define a healthy environment to create tailored, sustainable, and impactful interventions for improved health and wellbeing of people and planet.

Community-based participatory research and similar methods offer a helpful framework to facilitate synthesis of community views, such as those regarding interventions to improve community health and wellbeing [22,23]. However, the information synthesised from community members is only as rich as the engagement approach and will be limited and biased if not all parts of the community are involved. Engaging diverse communities has been shown to produce novel insights regarding what counts as an environmental issue, providing important research priorities [24]. Further, the intersectionality framework acknowledges that a multitude of factors influence marginalisation and builds a case for diversifying research participation to gain rich insights [25,26]. There is a need for creative approaches to community engagement in healthy environment research that is accessible and fit-for-purpose. Broomfield and colleagues (2021) advocate for atypical ways of engaging seldom heard community members, such as the use of pictures, videos, and interactive experiences, over traditional methods such as questionnaires and surveys [27]. Similarly, creative participatory research strategies have effectively engaged seldom heard groups, including ethnic minorities and youth, in a variety of topics, sparking conversation and community action [28,29,30]. These creative approaches to public engagement in research could pave the way for diverse perspectives of healthy environments to be understood and considered in political agendas.

The present study was set in the UK context where approximately 13.8% of the population belong to an ethnic minority group and 21% of working age adults have a disability [31,32]. Area-level deprivation is determined for each of the four nations of the UK independently—in England, 9.9% of the population live in the most deprived 10% of neighbourhoods [33].

This study aimed to understand whether the attributes that underrepresented groups of the UK public assign to healthy environments differ to those defined by the scientific community to date. The aim was achieved by holding focus groups with a purposive sample from across the UK. To obtain perspectives of people from underrepresented groups, these focus groups included people from ethnic minority groups and people with disabilities.

## 2. Materials and Methods

The reporting of this study, which was conducted from December 2020 to June 2021, is compliant with the COREQ checklist for qualitative research [34].

### 2.1. Participant Recruitment

In total, the study team aimed to recruit six co-creators and 100 participants, including 10 community hosts. Co-creators were involved throughout the study, having integral roles in its design and development of study materials. Community hosts recruited participants, completed a survey, watched videos, and facilitated focus groups. Participants completed a survey, watched videos, and shared their views in focus groups. Further details of the three different roles follow.

#### 2.1.1. Co-Creators

Co-creators provided expertise in environmental activism, research or policy during study design and development and assisted in ensuring all aspects of the study were accessible to the participants. Six co-creators were recruited in December 2020 through social media advertisements and organisations supporting public involvement across the UK that were known to the study team. In their applications, applicants shared their experience of research involvement and why they were applying for the role. Individuals were selected primarily on the basis that (1) they were experienced in environmental activism, research or policy and (2) they had a desire to increase public representation and involvement in research. A secondary priority was to recruit a diverse group of co-creators from different UK nations, of various ethnicities, ages, home environments, and genders. We also sought to recruit at least one co-creator who identified as having a disability. Over the course of the project, six monthly online meetings were held between the study team and the co-creators. Co-creators were paid to attend the meetings and for any preparatory work. Each meeting lasted approximately 90 min and open discussions were facilitated using interactive online software. The focus of these meetings included the community host and participant recruitment strategy, design of each of the project’s activities, and ensuring all terminology and activities were accessible to participants. Two co-creators additionally reviewed and approved the study’s findings and write-up.

#### 2.1.2. Community Hosts

Community hosts recruited participants to form ‘community groups’ which they facilitated in the focus group discussion. Ten community hosts were recruited through February and March 2021 by the study team, using purposive sampling, with selection based on their prior facilitation experience and connections to underrepresented community groups (pre-defined as people with disabilities, people living in more deprived areas, and people who are part of ethnic minority groups). Selection of the community hosts also accounted for diversity of age, gender, and UK geographical location. The involvement opportunity was advertised through established contacts who had access to experienced facilitators from underrepresented communities, including charities and co-production networks, and on social media (including Twitter and Instagram). A member of the study team spoke to each applicant to ensure they could conduct the role and communicate clearly in English since this was required for the transcription software. A few applicants were excluded on this basis.

#### 2.1.3. Participants

To adequately represent underrepresented groups of the UK public, the study team aimed to recruit 100 participants in total to form 10 distinct ‘community groups’, each led by a community host. Using a snowball sampling method, each community host formed a community group by recruiting nine participants from their own network. Community hosts were asked to prioritise recruitment of participants with disabilities, from ethnic minority groups, and living in more deprived areas, but due to the limited timeframe for recruitment participants were not excluded on this basis. The study team and co-creators provided an eye-catching, Plain English poster and email template for community hosts to share with their contacts. The key activities (including focus groups about healthy environments research), available support, and remuneration were summarised. Advertising materials also set out the criteria for participation: access to a smartphone and the ability to attend a focus group in April 2021. Participants also needed to be able to communicate their views to the community host by whom they were recruited—whether this was in English or another shared language (the community hosts would translate into English for the transcription software). Community hosts were supported by the study team throughout the recruitment process. Community hosts recruited the first nine applicants that met the inclusion criteria and passed their contact information onto the study team who followed up with the participant information sheet and consent form.

### 2.2. Study Design

This co-created qualitative study used a focus group run separately with 10 underrepresented groups from the UK public to encourage sharing of thoughts and feelings, through group discussion, on how certain outdoor environments made them feel and how they interpreted healthy environments and their key attributes. While the overall study framing and reporting uses ‘healthy environments’ in line with academic literature, the language used by community hosts during focus groups was ‘healthy spaces’. The co-creators encouraged the use of ‘healthy spaces’ based on it being more accessible to the public and more inclusive of built environments such as cities, whereas using ‘healthy environments’ could have inadvertently biased views to natural spaces. To prompt thinking about healthy environments in an immersive engagement experience, participants were sent a virtual reality (VR) headset and web links to a series of 360° videos which they watched on the VR headsets through their smartphones prior to the focus group. The 11 videos captured a range of outdoor environments in the UK, chosen by the study team and co-creators on the basis that they were most relevant to the UK public. These environments were: rivers, urban coastal, allotment garden, small urban green space, large urban park, windfarm, rural lakes, rural coastal, city, peatland bog, and industrial. Each video lasted approximately two minutes, with the last 20 s of the clip displaying an overlay of the question ‘Do you think this is a healthy environment and why?’.

### 2.3. Data Collection

At the start of the study, participants were sent an online survey to provide sociodemographic data (including gender, age, ethnicity, area of residence, languages spoken, and disability status). The focus groups took place from 19 to 26 April 2021, via the online platform Zoom, due to social distancing measures in place during the COVID-19 pandemic. Each of the 10 community hosts facilitated a 90 min focus group with their community group. Each group was attended by at least one study team member who took field notes and audio recorded the session. Some sessions were additionally observed by a co-creator and/or a member of the funding team.

A few participants communicated their views to the community host in their first language, for instance participants in one focus group spoke Bengali and the community host translated their views to English for the benefit of the study team member(s) and transcription software. Two community hosts each held separate discussions with one member of their community group who needed additional support, e.g., with the online technology and/or due to neural diversity. A palantypist attended two focus groups to live caption the discussion for participants with hearing loss.

The focus group facilitation guide, which was developed with and trialled by the co-creators, is provided in Appendix A. The facilitation guide was designed to address previously identified evidence gaps, including characterising how different social groups understand the potential of the natural environment to benefit human health [11]. To be more inclusive to the diversity of environments experienced by the participants and to not be overly prescriptive about what constitutes an environment, we kept the definition of ‘environment’ open to participants. This facilitated an increased richness in identifying how the environment can contribute to or detract from human health and wellbeing. Each community host started the focus group with an icebreaker, then confirmed that all participants had watched the 360° videos and completed the survey. The participants were then asked to reflect on their experiences of the 360° videos by sharing their responses to the following questions: “What did you like about the spaces you saw in the 360° VR videos? What didn’t you like? Why?” and “Has your view of a healthy space changed after exploring the 360° spaces? Why?”. The community host did not present participants with a definition of ‘healthy’ and instead left this open to interpretation to avoid biasing their views. However, it was made clear to participants that they should consider all outdoor settings or spaces. When asked for their views on healthy environments, participants were not explicitly asked to consider a healthy environment for any particular person, group of people, or for the environment itself. Respondents had the freedom to interpret healthy environments from their own perspective, allowing their own values or priorities to be expressed. To stimulate discussion, the community host could ask “How did these spaces make you feel?” or “If you could live in any of the 360° spaces presented, which would be your first choice? And your last choice? Why?” The community host continued to facilitate a discussion around these key questions until data saturation occurred where no new ideas were communicated by participants.

### 2.4. Data Analysis

Focus groups were audio recorded, transcribed verbatim, and imported into NVivo software (QSR, release 1.4.1 (851)), which facilitated transcript coding. Transcripts and audio recordings were stored in an access-controlled folder. The thematic analysis methodology was derived from Braun and Clarke (2012) [35]. Transcripts were coded during May 2021 by three independent reviewers (LR, OL, VA). The three reviewers listened to the focus group audio recording before reading the transcript and inductively assigned codes that pertained to the participants’ perceptions of factors constituting healthy environments. The first 10% of transcripts were coded in duplicate to ensure a consistent approach. The reviewers proceeded independently after confirming that pertinent passages from the transcripts were being coded in a similar manner and that the codes being generated could be understood independently of the raw data. Once all transcripts had been coded, the three reviewers combined their codes and sorted them into themes which were generated and revised through an iterative content analysis process [36] until a point at which all three reviewers were satisfied with the final result. The final themes represent the key views expressed by participants.

Area-level deprivation of participants’ place of residence was based on postcode, where provided, and calculated using the English, Scottish, or Welsh Index of Multiple Deprivation (IMD, SIMD, or WIMD).

### 2.5. Ethics Statement

Approval to conduct this study was granted by the Imperial College Research Ethics Committee (ICREC) and informed consent was obtained from all study participants. Participants were reimbursed for their time according to Involve Guidelines [37] and could withdraw both themselves and their data at any time.

## 3. Results

### 3.1. Co-Creator and Participant Characteristics

#### 3.1.1. Co-Creator Characteristics

Six co-creators based across England, Northern Ireland and Scotland were involved through study design and development. Their characteristics are set out in Table 1.

#### 3.1.2. Participant Characteristics

In total, 95 participants (including community hosts) were recruited and saw the project through to completion; of which 1% identified as non-binary, 38% male, and 61% female. 64% were from ethnic minority groups and 29% had a disability. Fifteen percent of participants were in the most deprived 10% of neighbourhoods in their country; generally, more participants were in the most deprived than the least deprived neighbourhoods. Six extra participants were initially recruited but dropped out of the study due to caring responsibilities or ill health and their data was removed.

Of the 10 community hosts, one was a young adult (18–25 years old), five had a disability and seven were people from ethnic minority groups. Six were female, three male and one non-binary. They represented each of the four nations of the UK by ethnicity or country of residence (England, Scotland, Wales, and Northern Ireland) and were aged 16–65. Demographic information for all participants, including community hosts, is set out in Table 2.

### 3.2. Key Themes

Our study found the following five themes to represent the most prominent contributors to or attributes of healthy environments as perceived by the diverse voices in our sample, listed in no particular order: (1) sounds and sights, (2) accessibility, (3) familiarity, (4) safety, and (5) mental health and wellbeing. Sounds and sights, accessibility, and safety were seen as indicators of a healthy environment; familiarity moderated the strength of the link between safety and healthy environments; and mental health and wellbeing was highlighted as a particularly important effect of a healthy environment on human health. These themes are described further in Table 3.

**Figure 1:**
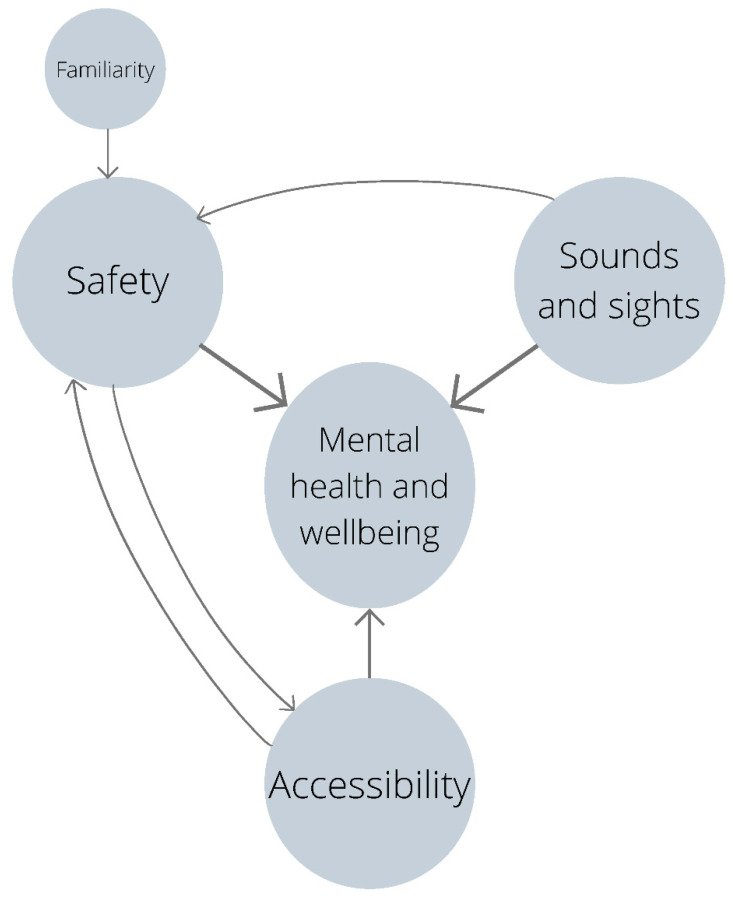
Visual representation of the links between the five key attributes of healthy environments highlighted by participants. The direction of the arrow infers the direction of influence.

These key themes are by no means independent of one another—their links, as agreed by the three reviewers, are demonstrated in Figure 1. These links were alluded to by participants during the focus groups and are highlighted within the description of each of the themes as follows. Participant quotes have been provided to illustrate each of these themes. Demographic information is provided for context in a manner that ensures no individual is identifiable.

#### 3.2.1. Sounds and Sights

Most participants cited sounds and sights as key indicators of a healthy or unhealthy environment. They were able to articulate how sensory elements of an environment made them feel, both physically and mentally, therefore linking the key themes ‘sounds and sights’ and ‘mental health and wellbeing’. There was a consensus that natural sounds, such as birdsong and crashing waves, make an environment feel healthier.

*“I realised the importance of the birdsong and where there was a variety of different birds singing … that kind of to me felt like it was a healthier space.”*

(female, urban home environment, 26–35 years old, white, self-reported disability and long-term condition)

*“The idea that the sound of your environment matters, which I don’t think I paid much attention to before, but that idea that birds are important, waves are important. You know, those kind of audio inputs are important.”*

(female, suburban/urban fringe home environment, 26–35 years old)

The sense of calm derived from the sounds of nature seemed to be amplified for some participants who lived in urban areas.

*“I like the sound of the birds and the lapping of the water … especially I think because I live in a crowded city.”*

(female, urban home environment, mixed heritage)

*“It [green and blue space] kind of gives you that break away from the hectic noise from, you know, if you’re in the city.”*

(female, urban home environment, 18–25 years old, self-reported disability)

The perception that healthier environments were those with fewer people around was common, and this was justified through the increased noise, road traffic and air pollution that crowds of people bring.

*“The peatland bog … that felt very healthy intrinsically. I guess it’s because there was no one around. What sort of stood out to me was, they [the 360° videos] didn’t show many people. There was no traffic. So they felt a lot healthier and … wilder and more peaceful. Then in the summer … loads of people, loads of noise, a lot of traffic going through the town … which feels kind of innately unhealthy.”*

(female, suburban/urban fringe home environment, 18–25 years old, white)

Certain sights inherent to an urban environment, such as smoke emitted from cars and little greenery, were associated with air pollution and negative consequences for physical health. This was particularly the case for those with health conditions that are exacerbated by air pollution, such as asthma. There was some concern that urban spaces, such as the one shown in the 360° video, are not designed to optimise human health in that they lack trees and other greenery.

*“Cities feel very stifling … there’s cars everywhere and there’s just a lot of noise and sound. Obviously, this is probably just biased because I have asthma, so I was automatically thinking ‘Oh no’. Even if I saw the smoke blooming out, it didn’t really make me feel too comfortable.”*

(male, urban home environment, 18–25 years old, African)

*“When I see the city [in the 360° video] … it’s not healthy at all. There’s loads of cars so pollution is definitely affecting everything … and there’s a lack of trees … there was only one type of shrub that I felt was just there for decoration.”*

(female, urban home environment, 18–25 years old, African)

Some participants, including those from urban areas, noted that the sounds of nature made them feel more connected to the wider natural world. Conversely, they felt disconnected from the natural world and even experienced poor mental health when they could hear noises of the city, such as from road traffic. This draws a link between the ‘sounds and sights’ and ‘mental health and wellbeing’ themes.

*“I’ve lived mostly in the inner city most of my life. And in terms of the videos, I felt most connected when I could hear the birds tweeting … Even though I live in an inner-city area, I really felt disconnected when I heard other noises [from the urban areas].”*

(female, urban home environment, Asian)

*“When we see the areas that we live in, you know, with all the traffic and stuff, that is what we see day to day. But then when you go out into the countryside, it’s really powerful. And you can breathe, and you can hear stuff. You can literally hear things, you know, birds and animals. In the city, all you hear is horns and cars and trucks. So it’s a bit depressing.”*

(male, urban home environment, Asian)

#### 3.2.2. Accessibility

The issue of accessibility was viewed through two lenses: geographical and logistical. Many participants, predominantly females, discussed the importance of geographical access, or proximity of their home, to different types of environments—such as urban or rural. Closely linked with the theme of ‘safety’, accessibility to nature in the logistical sense was of particular concern for participants with physical disabilities who required features that enable wheelchair access.

Viewing different types of environments of the UK through the 360° videos led participants to appreciate the green spaces they had access to in their own urban environments.

*“I was thinking of our town centre and we’ve got some trees there. And it [the small urban green space video] reminded me of that, and how lucky we are that there are trees in some of the shopping areas that we have in our city.”*

(female, coastal home environment, Asian)

For those with physical disabilities, some environments in the 360° videos were less accessible than others and viewing these provoked emotional responses. The beauty of remote environments, for instance the rural coast, was tarnished for participants who knew they would not be able to visit such places.

*“I had mixed reactions … it was beautiful, but that reaction was followed quickly with sadness and a sense of exclusion, because there’s likely no chance of getting to similar places in a wheelchair.”*

(female, urban home environment, 26–35 years old, white, self-reported disability and long-term condition)

An example of an environment displayed through the 360° videos that appealed to participants with physical disabilities was the peatland bog. It appeared to have a wide boardwalk that would facilitate people with all access needs and for this reason it was perceived as a desirable environment.

*“I noticed on the bog one … it had a nice accessible-looking boardwalk which is a really big deal in those kinds of areas, you know, otherwise they’re impossible.”*

(female, urban home environment, 26–35 years old, white, self-reported disability and long-term condition)

Making environments such as parks and rivers more accessible to people with physical disabilities was seen to also positively impact accessibility for the general population. Some participants agreed that people could be more likely to advocate and care for the natural environment if they are able to access it with ease—and this can in turn contribute to a healthier environment.

*“So accessible green and blue spaces … I use a wheelchair. And I think that if places are more accessible to people with disabilities, then they are more accessible to the average non-disabled person as well. And if you can get people into these spaces, they’re more likely to care.”*

(female, urban home environment, 26–35 years old, white, self-reported disability and long-term condition)

Considering features of healthy environments led participants to consider their ‘ideal’ healthy environment. Many found this challenged their preconceptions and biases—for instance, they found it difficult to label any of the environments presented in the 360° videos as completely ‘unhealthy’. Some participants voiced that, to function as a healthy society, we sometimes need to trade off typically ‘unhealthy’ aspects of an environment for accessibility and convenience but noted that healthy environments and environments typically associated with accessibility and convenience, such as urban areas, are not mutually exclusive.

*“It would be lovely to live away from the hustle and bustle of the busy areas that we are in, but we need a transport system.”*

(female, coastal home environment, Asian)

*“I realised that actually a healthy space and an urban environment … they aren’t mutually exclusive. You can kind of have both … when the 360° angle was kind of centred on the green space, I had a more in-depth look and realised that, despite the environment being in a city environment, in some cases, they were actually still healthy, which initially I kind of dismissed.”*

(male, suburban/urban fringe home environment, 18–25 years old, Asian)

#### 3.2.3. Familiarity

Participants’ memories and past experiences contributed significantly to their perceptions and biases toward or against certain environments. This seemed to be the case for females more than males and for people from ethnic minority groups more than the ethnic majority. While familiarity was not seen as a key attribute of a healthy environment, it moderated (strengthened or weakened) the link between safety and environmental health.

Those born outside of the UK had an affinity for environments that reminded them of their homeland, such as lakes, hills, or the coast. The location of participants’ home environments did not necessarily reflect this attraction to certain environment types—potentially due to trade-offs to be made between healthy environments and convenience, as expressed within the accessibility theme.

*“I’m quite a coastal person myself because my homeland is near the coast … so I love the water.”*

(female, urban home environment, Asian)

*“I really liked the lakes … that is one of my picks…I felt like it connected back to my homeland … you know like some of these hilly areas”*

(female, rural home environment, Asian)

Some participants shared views that familiarity lends itself to a sense of personal safety, comfort and security, which could provide an explanation for certain environments being preferred over others.

*“I’m from a countryside background. So it’s more familiar … when you’re familiar, you tend to be more safe”*

(male, 18–25 years old, suburban/urban fringe home environment)

Familiar associations or stereotypes also played a part in participants’ perceptions of certain environments as unhealthy. Familiarity may have been fostered through personal experiences of those places, or otherwise indirectly through seeing or hearing of events occurring in those environments on the news, television programmes and other sources. For example, areas with train tracks were viewed as places where crimes took place, and cooling towers were associated with pollution and breathing problems.

*“I know the urban coastal area will have problems with pollution, I just know it. You can’t see it in the video, but I know that to be the case because the boats discharge and the fishing industry discharges as well, so they’re bound to.”*

(Female, suburban/urban fringe home environment, white, self-reported long-term condition)

Conversely, when viewing environments through the 360° videos that were unfamiliar, a few participants tended to view those environments negatively, further demonstrating the moderating effect of familiarity on perceptions of safety and healthy environments.

*“When it came to the seafront or coastal views, it was unfamiliar and very quiet. And if I were in that space, I wouldn’t really know how to navigate it. And feel really unsafe in that space as well.”*

(Female, urban home environment, 18–25 years old, African)

#### 3.2.4. Safety

For a healthy environment, safety was a primary concern of participants from a range of community groups, but particularly females and those who had disabilities and/or long-term conditions. Safety was associated with three distinct factors: healthy air, reduced risk of physical harm from accidents (e.g., road traffic accidents), and freedom from physical violence. Often these factors were in competition and thus views varied regarding which of the environments portrayed in the 360° videos appeared to be safest. For example, many participants considered safe aspects of rural environments to be healthy air and reduced risk of road accidents, yet some participants, including those with disabilities and from ethnic minority groups, characterised urban areas as safe environments due to the number of people around at all times.

With more people around, participants felt safe as a result of ‘blending in’ to a crowd. This was particularly the case for participants from ethnic minority groups or with a disability/long-term condition.

*“I like urban green spaces because I like people around, and I feel safer with people around.”*

(female, suburban/urban fringe home environment, white, self-reported disability and long-term condition)

*“I love the countryside, but also know that I’m probably not going to see another black or brown person and feel people are looking at me. Whereas in the city I very much enjoy the feeling of just walking around and feeling, you know, blending in …”*

(female, mixed heritage)

This sense of ‘safety in numbers’ conflicts with the negative association between common urban sounds (e.g., of road traffic) and sights (e.g., of air pollution from car exhaust fumes), and physical and mental health and wellbeing. The notion that safety in numbers makes an environment preferable also conflicts with the ‘sounds and sights’ theme whereby environments with noisy crowds of people were described as “innately unhealthy”. This illustrates a tension between different aspects of the same environment that may contribute to or detract from health and wellbeing.

*“It probably is not a physically healthy space pollution-wise, but I always feel safest in the city.”*

(female, urban home environment, 36–45 years old, white, self-reported disability and long-term condition)

Some participants, of varying ethnic backgrounds, felt safer in rural environments than in cities due to the green spaces and reduced road traffic, reducing air pollution and the risk of cycling accidents. A few participants chose to raise their family in rural environments due to them being perceived as safer and therefore healthier, despite needing to trade-off these benefits with reduced accessibility to other needs, such as their workplace.

*“I like to go there [the city] for work, and then come back, but I like to leave that behind. I think when you have kids … you like to come back to somewhere that you feel like they are safe in terms of, you know, the green space, and then the pollution as well.”*

(female, urban home environment, 36–45 years old, African)

Similarly, the 360° video of an industrial environment made a few participants feel explicitly unsafe. This environment was also perceived as unhealthy due to the air pollution which could negatively impact the health of people living or working nearby.

*“You know that the industry one … with the lorries and the traffic. It kind of made me feel unsafe … it just didn’t feel comfortable”*

(female, urban home environment, 36–45 years old, white, self-reported disability and long-term condition)

*“The industrial side … the quality of the air could be a lot worse … there’ll be more pollution, and it might not be as healthy for you as, say, you lived and worked in a rural environment.”*

(female, 18–25 years old, White and Asian)

#### 3.2.5. Mental Wellbeing

For many participants, particularly adolescents and those from ethnic minority groups, a healthy environment was seen as an environment that had a positive effect mental health and wellbeing. In turn, ‘unhealthy’ elements of an environment, such as loud noises or air pollution, were found to be overwhelming and distressing. A majority of participants believed the environments that had the most positive impact on mental health and wellbeing were the rural, open environments, near coastal or blue spaces.

*“I think they’ve [open green and blue spaces] had such an impact on my mental health so positively.”*

(male, suburban/urban fringe home environment, 18–25 years old, Asian)

*“I realised my mental health is much better when I’m in Bangor, even though it is boring and there’s not much to do. But I can go out and see the golf course; I can go to Bangor Mountain; I can go to the pier and seeing all that greenery, the water, the birds. It does something to me mentally.”*

(female, urban home environment, self-reported disability)

Participants who grew up in rural areas with vast open spaces commonly cited feeling overwhelmed when entering the city.

*“Being out in a green space really helped my mental health. Where I live, it’s quite green, quite quiet. It’s not urban at all. And when I go to university in London it’s really overwhelming for me.”*

(female, 18–25 years old)

Among city dwellers, vast, open spaces in urban environments were a good substitute for the relaxing attributes of rural environments. This sense of space in an otherwise busy city supported physical and mental health through providing relaxation.

*“You could see for a good few hundred metres … even if it was an urban environment, as long as you weren’t kind of closed in by buildings and stuff like that … as long as you feel you have some kind of space in front of you, then it can lead to a person feeling more relaxed and, in turn, maybe being slightly more healthy.”*

(male, urban home environment, 36–45 years old, self-reported long-term condition)

For many, accessible and quiet green and blue spaces were seen as a refreshing safe haven from the stressors of daily life. This notion draws a link between four of the key themes: ‘accessibility’, ‘sounds and sights’, ‘safety’, and ‘mental health and wellbeing’.

*“I was feeling a bit stressed at work. So after, you know, I’m going to log off, and get my bicycle. And I’ll just go around for 30 min. And I felt great … just looking at the green spaces, taking it all in … I felt refreshed. So it’s something like that. Just having that peace around and feeling safe.”*

(female, urban home environment, 36–45 years old, Asian)

Social connection and a sense of community were aspects of an environment which were considered healthy through their positive effect on mental health and wellbeing. These environments could be buildings, such as places to practice religion, or outdoors, such as in community gardens.

*“That’s why they’re [mosques and churches] important because that’s how you form community and shared values. Different studies talk about how some sense of spirituality keeps people happy in life, just being able to connect, whether it’s to your surroundings or to what you believe to be a higher power.”*

(male, urban home environment, African)

*“Like community gardens, I think that’s also not just good for the environment, but it has an impact on mental health because you can go, and you can have your chill out time. You can speak to people, you can socialise.”*

(female, rural home environment, 26–35 years old, white)

Links were drawn between key themes ‘accessibility’ and ‘mental health and wellbeing’, with a particular focus on inclusive societies. A few participants suggested that a healthy environment is one that is inclusive of all people and their views. As such, people that are socially isolated and unable to communicate their views will have poorer mental health and wellbeing.

*“Inclusive community … in terms of healthy space … without everybody being included and being recognised, I think that spaces become negative because … there’s the psychological impact of being stressed, of being unable to communicate, to change. People are less likely to be aggressive or … domestic violence and things like that are reduced … and also depression—if there’s things for them to do and communities to go to …”*

(female, urban home environment, white, self-reported disability and long-term condition)

Some participants framed their view of a healthy environment by describing elements that they preferred or that had the greatest impact on their wellbeing. However, it was recognised that healthy environments are not just for human health, but that the health of flora, fauna, and the wider ecosystem should also factor into definitions.

*“My feelings changed about what a healthy environment was … I started off with what makes me feel nicest, what I prefer … like accessibility, public transport … but then I remember my impression of the peatland bog [in the 360° video] is very healthy because there’s lots of insects, lots of life and rotten things … but that doesn’t mean that it was my favourite place … so maybe a healthy environment isn’t just about how I feel about being there.”*

(female, mixed heritage)

## 4. Discussion

This study engaged underrepresented groups of the UK public to understand the attributes they assign to healthy environments. Through engagement with these groups, we collated perceived key attributes of healthy environments, including safety (and its moderator, familiarity), accessibility, and sounds and sights. The key aspect of human health raised by the groups in their discussion of healthy environments was mental health and wellbeing. Some of these attributes have previously been considered in environmental research, while this study also identified novel facets of healthy environments that are important to underrepresented communities but that are not commonly considered by national and global health organisations [2,4,5].

Pre-existing definitions of a healthy environment, such as from the World Health Organization, are limited in that they were devised by experts, not members of the public. Therefore, they likely capture only the views of more privileged socioeconomic groups and exclude the considerations of marginalised populations such as people from ethnic minorities, those living in more deprived areas, and people with disabilities. While pre-existing definitions largely focus on physical health benefits of healthy environments, our study found a healthy environment can also benefit mental health and wellbeing. Individuals’ past experiences and familiarity with certain environments, as well as the areas in which they live, seem to strongly influence their views of healthy environments. Involving participants with physical disabilities meant accessibility was an important feature of a healthy environment, with urban environments largely considered more accessible than rural environments. How safe an environment was perceived to be influenced perceptions of healthy environments, particularly of participants with disabilities/long-term conditions, or those from ethnic minority groups. This also impacted how likely participants were to access certain environments. Sounds and sights were strong drivers of many participants’ views of what was healthy for their physical and mental health and wellbeing, as well as for the environment itself.

Our research highlighted the importance of nature to the public, not just as the preserve of rural or countryside areas, but also in urban areas. Those who lived in or visited busy cities appreciated natural green and blue spaces within urban areas for providing a refuge that is essential for mental health and wellbeing. Some of the focus groups discussed the distinction between what is healthy for the non-human world versus what is a healthy environment for people. A healthy environment for people was considered one that is inclusive and accessible to all members of society, whereas natural environments that were free of people were viewed as healthier for other species. Previous research involving adolescents reported accessibility as the only positive feature of an urban environment [38], yet our study additionally found urban environments could have positive attributes of familiarity and personal safety. Our participants also reflected on how green spaces in cities are beneficial to their mental health and wellbeing, and a minority specified that it is the sense of space or vastness that facilitates this benefit. This sentiment echoes findings from Sharifi, Nygaard, and Stone (2021) that only larger (than about 9064 m^2^ or 2.2 acres) urban green spaces have a significantly positive association with wellbeing [6], building a case for green corridors and green walls that can create the illusion of an enlarged green space.

While not a key feature of previous healthy environment definitions, sounds and sights of an environment have previously been linked to human health—such as the ability of birdsong to reduce stress and improve mood [39,40]. Similarly, noise pollution has previously been found to negatively impact quality of life [41] and increase susceptibility to hypertension and cardiovascular disease [42].

Expert definitions of healthy environments perceive safety as preventing communicable and non-communicable disease by minimising environmental hazards [2,4,5]. While our study confirmed the public are concerned with safety regarding air quality and its impact on health, they also valued busy cities for feeling safe from interpersonal harm and blending in by being among crowds. These two aspects of safety are defined as ‘Health security’ and ‘Personal security’, respectively, in the Safe Cities Index [43]. Busy cities were perceived by our participants to increase personal security (‘safety in numbers’) but decrease health security (due to air pollution) implying that in urban environments these various aspects of safety can conflict. This indicates that current definitions of a healthy environment may need to be expanded to accommodate various interpretations of safety from diverse groups.

Just as tensions exist between these different aspects of safety and their impacts on healthy environments, tensions exist between key themes. This implies that there is no one ideal healthy environment or that one environment may not be entirely healthy. For instance, attributes of some urban environments that were considered health-promoting were safety derived from crowds, familiarity, and ease of access for all people, whereas attributes seen as a hindrance to good health were stress-inducing noise, air pollution, and the lack of natural sounds.

This study has several strengths. First, it presents an expanded understanding of what constitutes a healthy environment that is needed to inform public policies that impact the environment in which people live. The public views comprising this updated understanding were not biased by the study team providing any predetermined definitions of what a healthy environment is. Instead, participants were asked to share their own views, influenced only by their unique experiences and context. The diversity of participants across different ages, disability statuses, ethnicities, and from different home environments (such as urban, rural, but also area-level deprivation) increased the richness of the findings by providing an intersectional perspective [25,26]. Inequalities are exacerbated when policy is based on studies that are biased towards the majority [1]. Our study avoided this bias by listening to voices from underrepresented communities that are seldom heard in research. Visual research methods can be effective at describing the ineffable [44]. As such, our study’s immersive approach, using 360° videos and VR headsets, helped participants to visualise different environments and meant their views were not solely influenced by their immediate surroundings. In devising resourcing plans, it is important for other researchers to acknowledge that some participants may need extra support to engage with technology.

Co-production can benefit research by supporting participant-centric decision-making to optimise accessibility and inclusivity of diverse voices. Involving members of the public benefits research through sharing of skills, knowledge and social networks [45]. This can involve enhancing accessibility of the study to participants by limiting bias and complex language [46]. Co-production of this study with members of the public ensured underrepresented groups were recruited; optimised accessibility of the activities and language used; and ensured the activities were engaging. The novel study methodology can be replicated for public engagement research that aims to involve and engage diverse or underrepresented groups. The snowball sampling strategy enabled engagement with communities that are less represented in research—it is important to engage these communities in healthy environments research to avoid widening existing inequalities [4]. Sharing a first language and similar background to their community host and other members of the focus group led to participants building trust and rapport quickly. Having the focus groups led by a community host, rather than a researcher, helped to diminish power dynamics, creating a relaxed and comfortable environment that encouraged sharing of ideas and stories. While typically online activities are reported to exclude some groups of society, our participants preferred the online study environment as it removed the requirement to travel. This improved accessibility for participants with physical disabilities and those who live in locations further away from those conducting the study.

This study also had some limitations. Access to a stable internet connection, a smartphone, and Zoom video conferencing software were prerequisites for involvement in the study and may have excluded some participants. To counteract this, community hosts were encouraged to support participants with technology as needed. While most participants enjoyed viewing the 360° videos, the VR headset was not universally suitable. A few participants found the headset incompatible with wearing glasses or inducing dizziness. The study involved participants from three of the four UK devolved nations, yet none were from Northern Ireland. Participants from Northern Ireland may have had unique views due to the country’s distinctive cultural and political history. Further, due to the limited number of applications for the co-creator role, only one co-creator identified as part of an ethnic minority group. A member of the study team attended each focus group as a silent observer, and this meant they were not able to probe on certain points of interest. This meant the study team was limited during analysis and interpretation in differentiating between the attributes that participants thought were healthy for them, healthy for other people, and healthy for wider ecosystems. Of note, this study does not claim to be representative of underrepresented groups in the UK due to the limited sample and heterogenous nature of sub-populations such as people with disabilities. These groups cannot be assumed homogenous and significant intra-group variation exists. The area-level deprivation score, IMD, SIMD or WIMD for England, Scotland and Wales respectively, provides information about the area in which the participants lived, but cannot be extrapolated to the level of the individual. However, the additional attributes of healthy environments perceived by participants in our study beyond those typically outlined in academic literature serve a key purpose, namely to highlight the need to diversify perspectives on the interconnections between human health and a wide range of environments.

Co-benefits of climate action are increasingly cited in governments’ climate change agendas [47,48]. These are the additional benefits that emerge from policies primarily aimed to reduce greenhouse gas emissions, such as benefits to human health resulting from reduced air pollution associated with the shift away from petrol and diesel vehicles. Going forward, there is a need for climate action to be aligned with efforts to protect and enhance the health of people and nature, and to do so in a way which is culturally sensitive and inclusive of different people’s perceptions of a healthy environment. Understanding how the public views healthy environments and the environmental attributes that are important to them, as well as the trade-offs they deem acceptable, could help shape how these policies can be prioritised, communicated and implemented to maximise public support and involvement. The understanding of underrepresented groups’ views in these areas can also inform how these groups can be encouraged to participate in biological conservation and environmental sustainability programmes. Our findings suggest people might hold back from accessing environments that are unfamiliar, make them feel unsafe, will make them stand out, or exacerbate physical accessibility issues. Further, our findings highlight the importance of fostering inclusivity through ensuring physical accessibility to all public spaces and for all public members. This inclusivity can foster supportive attitudes towards the environment by all public members.

While some participants were able to articulate what they considered to be a healthy environment for people or for non-human nature, to many this distinction was not so clear. This inability to separate what is healthy for non-human nature and what is healthy for people builds the case for the One Health approach to healthy environments research and policy [49,50] which, through multisectoral collaboration, appreciates that humans, animals, and the environments in which they live are all intrinsically linked. The complex nature of healthy environments also supports the need for a health in all policies approach [51], whereby non-health sectors consider the effect of their policies and actions on the health of people and the wider ecosystem.

In addition to surfacing novel attributes of healthy environments, this study revealed insights into what participants did and did not like about an environment. We found that there can be a tension between preferences for an environment and perceptions of its value to human health. Future research could look to distinguish between these two concepts since understanding both can be useful for policy. Large-scale quantitative survey studies could build on this study’s findings to explore their generalisability and differentiate between sub-populations, for instance by focusing on specific groups such as people from lower socioeconomic backgrounds. These studies should explore the key attributes of healthy environments identified in this study in more depth to uncover the underlying values and reasons for these perceptions. Future research could also aim to distinguish between what the public believe are attributes of a healthy environment for themselves as individuals, their communities, and the natural world itself—and how these concepts are highly interdependent. Since this is the first known study of its kind, it would be beneficial for other researchers to replicate our study with a larger sample of underrepresented groups including people with disabilities or long-term conditions, people from lower socioeconomic backgrounds, and people from ethnic minority groups, to better understand its generalisability and compare across subgroups.

## 5. Conclusions

In conclusion, this study identified key attributes of healthy environments as perceived by an underrepresented sample of the UK public: (1) sounds and sights, (2) accessibility, (3) familiarity, and (4) safety. Mental health and wellbeing was identified as a particularly important aspect of human health that the environment can affect. These findings bring a new perspective to existing definitions that are typically limited to impacts of the environment on public physical health, and have to date not fully considered how diverse communities may differentially ascribe human health benefits to different environments. While environmental research has focused mostly on rural natural environments, this study also highlighted the importance of built urban environments for mental health and wellbeing, based on their perceived attributes of safety and familiarity. Nature-based urban environments can contribute to better mental health and wellbeing of underrepresented communities provided they foster inclusivity by being accessible to all. In line with previous research, sounds and sights were found to influence how healthy an environment is perceived to be. This body of work can provide a people-centred evidence base for policies that impact the environments in which people live and better accounts for the diversity of perspectives across the community.

## Figures and Tables

**Table 1:** Characteristics of co-creators as self-reported using an online survey.

Co-Creator Characteristic		Count/6
Age	16–25	3
	26–45	1
	46–65	2
Gender	Female	4
	Male	2
Ethnicity	White	3
	White Irish	1
	White Other	1
	Pakistani	1
Country of residence	England	4
	Northern Ireland	1
	Scotland	1
Home environment	Urban	2
	Rural	2
	Urban fringe	1
	Coastal	1
Disability	Yes	1
	No	5

**Table 2:** Characteristics of study participants as self-reported using an online survey.

Participant Characteristic		Count/95 (%)
Age	16–25	26 (27%)
	26–45	50 (53%)
	46–65	14 (15%)
	66–75	5 (5%)
Gender	Female	58 (61%)
	Male	36 (38%)
	Non-binary	1 (1%)
Ethnicity	White	34 (36%)
	African	20 (21%)
	Bangladeshi	15 (16%)
	Mixed	6 (6%)
	Pakistani	6 (6%)
	Indian	5 (5%)
	White Irish	3 (3%)
	Kashmiri	2 (2%)
	Asian (Other)	1 (1%)
	Arabic	1 (1%)
	Malay	1 (1%)
Country of residence	England	75 (79%)
	Scotland	10 (10.5%)
	Wales	10 (10.5%)
Home environment	Urban	60 (63%)
	Urban fringe	22 (23%)
	Rural	8 (8%)
	Coastal	5 (5%)
Index of Multiple Deprivation (IMD)—1 is most deprived decile; 10 is least deprived decile	1	13 (15%)
	2	10 (12%)
	3	9 (11%)
	4	13 (15%)
	5	5 (6%)
	6	11 (13%)
	7	8 (9%)
	8	9 (11%)
	9	3 (4%)
	10	4 (5%)
Disability	Yes	28 (29%)
	No	63 (66%)
	Prefer not to say	4 (4%)
Caring responsibilities	Yes	24 (25%)
	No	69 (73%)
	Prefer not to say	2 (2%)

**Table 3:** Key themes and associated quotes.

Theme	Description	Example Quotes
Sounds and sights	Green space and birdsong were sensory aspects that made an environment seem healthier; environments without greenery and with sounds of road traffic were perceived as less healthy.	*“I realised the importance of the birdsong and where there was a variety of different birds singing … that kind of to me felt like it was a healthier space*.” *“When I see the city [in the 360° video] … it’s not healthy at all. There’s loads of cars so pollution is definitely affecting everything … and there’s a lack of trees … there was only one type of shrub that I felt was just there for decoration.”*
Accessibility	Geographical and logistical accessibility were important aspects of a healthy environment. The former relates to the proximity, such as of nature, to communities; the latter is about physical aspects of an environment that can make it more or less accessible to certain communities. Logistical accessibility was of particular concern to participants with physical disabilities whose access to certain environments can be limited by physical barriers.	*“It would be lovely to live away from the hustle and bustle of the busy areas that we are in, but we need a transport system.”* *“I had mixed reactions … it was beautiful, but that reaction was followed quickly with sadness and a sense of exclusion, because there* *’s likely no chance of getting to similar places in a wheelchair.”*
Familiarity	Familiarity moderated the strength of the link between safety and healthy environments. Environments that were most familiar to participants were often where they felt safest. Hence these environments improved mental health and wellbeing and were considered healthier than unfamiliar environments.	*“I’m from a countryside background. So it* *’s more familiar … when you’re familiar, you tend to be more safe”* *“When it came to the seafront or coastal views, it was unfamiliar and very quiet. And if I were in that space, I wouldn’t really know how to navigate it. And feel really unsafe in that space as well.”*
Safety	The link between safety and healthy environments was perceived in three respects: healthy air, reduced risk of physical harm from accidents (e.g., road traffic accidents), and freedom from physical violence. These aspects were seen to compete with each other by some participants who, for instance, felt safer from physical violence in cities where the air is less healthy.	*“I like urban green spaces because I like people around, and I feel safer with people around”* *“The industrial side … the quality of the air could be a lot worse … there’ll be more pollution, and it might not be as healthy for you as, say, you lived and worked in a rural environment.”*
Mental health and wellbeing	Mental health and wellbeing was often cited as a primary outcome that a healthy environment can have for people. Often, when referring to a ‘healthy environment’, participants reflected on the benefits to their mental health and wellbeing.	*“Being out in a green space really helped my mental health. Where I live, it’s quite green, quite quiet. It’s not urban at all. And when I go to university in London it’s really overwhelming for me.”* *“You could see for a good few hundred metres … even if it was an urban environment, as long as you weren’t kind of closed in by buildings and stuff like that … as long as you feel you have some kind of space in front of you, then it can lead to a person feeling more relaxed and, in turn, maybe being slightly more healthy.”*

## Data Availability

Not applicable.

## Appendix A

**Table A1:** Focus group facilitation guide.

Activity	Detail
Community host introduces self, the focus of the session, and gives participants the opportunity to introduce themselves.	Talking points: Welcome Housekeeping Set the tone—everyone’s opinion is valuable, no silly questions, listen to each other Explain icebreaker Run through agenda Mention we are recording the meeting for note taking purposes
Community host facilitates icebreaker	Please say your name, location, and 3 words to describe the environment you live in
Community host leads a conversation on the group’s reflections after watching the 360 videos (before the workshop).	What did you like about the spaces you saw in the 360 Virtual Reality videos? What didn’t you like? Why? How did these spaces make you feel? If you could live in any of the 360 spaces presented, which would be your first choice? And your last choice? Why? Has your view of a healthy space changed after exploring the 360 spaces? Why?
